# Fear of Cancer Recurrence Prevalence and Its Associated Factors Among Family Caregivers of Women With Breast Cancer: A Systematic Review and Meta‐Analysis

**DOI:** 10.1111/jocn.17680

**Published:** 2025-02-24

**Authors:** Xiaofan Bu, Ling Jiang, Doris Y. P. Leung

**Affiliations:** ^1^ School of Nursing The Hong Kong Polytechnic University Kowloon Hong Kong; ^2^ Department of Nursing The Affiliated Suzhou Hospital of Nanjing Medical University Suzhou China

**Keywords:** breast cancer, caregivers, fear of cancer recurrence, risk factors, systematic review

## Abstract

**Background:**

The fear of cancer recurrence (FCR) levels reported by caregivers are as high as those reported by women with breast cancer, with some caregivers even reporting FCR levels higher than women with breast cancer. The recognition of factors associated with caregiver FCR is important for providing proactive support to caregivers at risk.

**Objective:**

To identify factors associated with high FCR in caregivers of women with breast cancer.

**Methods:**

A systematic search of eight electronic databases was conducted from database inception to August 2023. The identified papers were screened, and their full texts were further assessed. The quality of the included studies was examined by using a checklist, and relevant data were extracted with a predeveloped data extraction form. The best‐evidence synthesis model was used for data synthesis. Meta‐analysis was conducted to calculate the prevalence of caregiver FCR.

**Results:**

The search yielded a total of 2137 studies, and 15 studies involving 2461 caregivers were included after the screening and full assessment of 56 papers. A total of 29 factors were identified. Of these factors, five factors with a moderate level of evidence associated with high FCR were identified: insufficient communication of women with breast cancer, low level of resilience, high social constraints, high protective buffering and insufficient communication of caregivers; 15 associated factors were supported by limited‐level evidence and nine were supported by conflicting‐level evidence. The prevalence of FCR in caregivers was 45%.

**Conclusions:**

The associated factors examined provide some evidence for identifying caregivers who are at high risk of high FCR. Identifying factors contributing to FCR in caregivers is important for developing interventions for those caregivers most in need and reducing adverse health outcomes related to caregiver FCR. Additional studies are needed to examine the relationship between conflicting factors and caregiver FCR.

**Patient or Public Contribution:**

No patient or public contribution.

**Trial Registration:** PROSPERO registration number: CRD42023469754; identifier: https://www.crd.york.ac.uk/PROSPERO/#recordDetails

**Summary**

- What does this paper contribute to the wider global clinical community?
  ○ FCR was commonly experienced by caregivers of women with breast cancer, showing a prevalence of 45%.
  ○ Caregivers with low resilience, high social constraints, high protective buffering and insufficient communication tended to experience higher caregiver FCR than those without.
  ○ Caregivers tended to experience higher FCR than other caregivers if they had less communication with women with breast cancer.

## 1 Introduction

From 1990 to 2019, the incidence of breast cancer increased by 128.32% (Xu et al. 2021). The number of newly diagnosed breast cancer cases is projected to grow by over 40% to approximately 3 million cases every year by 2040. Breast cancer accounts for one in four cancer cases and one in six cancer deaths amongst women, ranking first in terms of cancer incidence in the majority of countries (Sung et al. 2021). Deaths from breast cancer are expected to increase by more than 50% to approximately 1 million in 2040 (Arnold et al. 2022).

Caregivers experience the challenges of living with uncertainty and unanswered questions on a daily basis (Traboulssi et al. 2022). Additionally, they fear the malignancy of cancer and its possibility to advance and recur as another form of cancer (Younes Barani et al. 2019; Zhu et al. 2012). They feel as if they are living with a high degree of uncertainty (Traboulssi et al. 2022; Zhu et al. 2012). This uncertainty is not limited to concerns about the distant future; it also includes concerns related to the disease, treatment outcomes and expectations on a day‐to‐day basis, making caregivers a vulnerable group requiring professional support (Fitch and Allard 2007; Traboulssi et al. 2022).

Fear of cancer recurrence (FCR) is a top‐ranked unmet need of cancer caregivers. It refers to fear, worry or concern about cancer returning or progressing (Lebel et al. 2016). Evidence from systematic reviews supports FCR as a prevalent unmet need of cancer caregivers (Smith et al. 2022; Webb et al. 2023). A systematic review by Webb et al. reported that approximately 48% of caregivers of patients with cancer have clinically significant FCR (Webb et al. 2023). Another systematic review by Neves et al. showed that over half of caregivers reported moderate‐to‐high levels of FCR (Neves et al. 2023). Recently, cross‐sectional studies have demonstrated that between 19% and 64.95% of caregivers of women with breast cancer had clinically significant FCR (Janz et al. 2016; Perndorfer et al. 2019; Soriano et al. 2021; Wang et al. 2021). Another study found that FCR in caregivers of women with breast cancer was higher than that in patients themselves (Janz et al. 2016).

Some studies have reported the consequences of FCR in caregivers of patients with cancer. Caregivers' FCR is related to poor adjustment, cancer‐specific stresses/concerns, quality of life and emotional/mental functioning (Smith et al. 2022). Moreover, it may worsen outcomes in patients with cancer, either by reinforcing patients' concerns about recurrence or by compromising caregivers' ability to support patients (Smith et al. 2022). Studies have found that caregiver FCR is associated with psychological morbidity and impaired quality of life in patients, caregivers and family (Smith et al. 2022).

An increasing number of studies have been conducted to measure FCR in caregivers of women with breast cancer or explore multidimensional factors associated with their FCR. Low educational levels, social support and resilience and high social constraints have been identified to be associated with high FCR in caregivers of women with breast cancer (Cohee et al. 2017; Perndorfer et al. 2019; Soriano et al. 2021; Soriano, Pasipanodya, et al. 2018; Wang et al. 2021; Zhang, Yu et al. 2023). However, thus far, no systematic review on the associated factors of FCR in caregivers of women with breast cancer has been found. The identification of factors associated with increased FCR in caregivers is important for the development of strategies for reducing the risk of FCR in caregivers. Therefore, we performed a systematic review to examine the associated factors of FCR in caregivers of women with breast cancer and grade the evidence in accordance with the quality of the reviewed studies. Furthermore, we conducted a meta‐analysis on the prevalence of caregiver FCR. Our review question is ‘What are the associated factors of FCR in caregivers of women with breast cancer and what is the prevalence of caregiver FCR’?

## 2 Methods

### 2.1 Design

This systematic review was conducted and reported in accordance with the Preferred Reporting Items for Systematic Reviews and Meta‐Analysis 2020 statement (Page et al. 2021). It was registered in the International Prospective Register of Systematic Reviews in 2023 (CRD42023469754).

### 2.2 Literature Search

A thorough literature search was conducted on 8 and 9 August 2023 by using eight databases, including PubMed, Web of Science, CINAHL, Ovid, EMBASE, CNKI, Wangfang and Sinomed. Duplicate articles were removed, and the two authors independently screened titles, abstracts and full texts until agreement regarding the final articles for inclusion was reached. Table 1 shows the search strategy in PubMed.

**TABLE 1:** Search strategy in PubMed.

1	(‘breast’[MeSH Terms] OR ‘breast’[Title/Abstract] OR ‘mammary’[Title/Abstract]) AND (‘neoplasms’[MeSH Terms] OR ‘carcinoma’[MeSH Terms] OR ‘tumour*’[Title/Abstract] OR ‘carcinoma*’[Title/Abstract] OR ‘neoplasm*’[Title/Abstract] OR ‘cancer*’[Title/Abstract])
2	‘fear’[MeSH Terms] OR ‘anxiety’[MeSH Terms] OR ‘fear’[Title/Abstract] OR ‘afraid’[Title/Abstract] OR ‘anxi*’[Title/Abstract] OR ‘worr*’[Title/Abstract]
3	‘back’[MeSH Terms] OR ‘recurrence’[MeSH Terms] OR ‘disease progression’[MeSH Terms] OR ‘disease free survival’[MeSH Terms] OR ‘back’[Title/Abstract] OR ‘progress*’[Title/Abstract] OR ‘recur*’[Title/Abstract] OR ‘relaps’[Title/Abstract] OR ‘reoccur*’[Title/Abstract] OR ‘return*’[Title/Abstract] OR ‘spread’[Title/Abstract]
4	‘caregivers’[MeSH Terms] OR ‘spouses’[MeSH Terms] OR ‘carer*’[Title/Abstract] OR ‘caregiv*’[Title/Abstract] OR ‘care give*’[Title/Abstract] OR ‘famil*’[Title/Abstract] OR ‘parent*’[Title/Abstract] OR ‘spouse*’[Title/Abstract] OR ‘partner*’[Title/Abstract] OR ‘husband*’[Title/Abstract] OR ‘child*’[Title/Abstract]

### 2.3 Study Selection and Eligibility Criteria

Original observational studies that assessed associations between caregiver FCR with at least one variable by using correlation, comparison or regression analysis were selected. Studies published in the English or Chinese language were considered in this review if they met the following inclusion criteria:

1. Types of participants: caregivers of women with breast cancer, such as any relative, partner, friend or neighbour with an important personal relationship to the women with breast cancer.
2. Study design: cohort, longitudinal and cross‐sectional studies.
3. Condition: caregiver FCR should be the primary or secondary outcome measured at any course of the cancer trajectory, with at least one associated factor reported using a validated or non‐validated instrument.
4. Context: studies conducted in any setting, including homes, hospitals (inpatients and outpatients) and any other appropriate location considered to have a general understanding of the context.

A study was excluded if it met any of the following criteria:

1. The study shared the same sample with another major study included in the review.
2. Caregiver FCR was an independent variable instead of an outcome.
3. Studies included mixed cancers without subgroup analysis for caregivers of women with breast cancer.
4. Studies without full texts.

### 2.4 Selection

We imported records from searches into an EndNote library (EndNote X9.1), and duplicate studies were removed. The remaining records were transferred to an Excel spreadsheet (Microsoft). Screening was conducted by two independent reviewers who assessed the titles, abstracts and full texts of articles. Articles that did not meet the established eligibility criteria were excluded. Any disagreement between the two reviewers was resolved by discussion or in consultation with other investigators.

### 2.5 Quality Assessment

The methodological quality of the included studies was independently assessed by two reviewers by using a modified checklist adapted from Ge and Mordiffi (2017) and Zhao et al. (2015). The checklist contained five aspects to be assessed by nine individual criteria (Table 2). These checklist criteria were designed for the methodological quality assessment of observational studies and have been used in previous observational systematic review articles (Ge and Mordiffi 2017; Gomes and Higginson 2006; Xing et al. 2013; Zhao et al. 2015; Zhou et al. 2013). Disagreements in quality assessments were resolved through discussion. No point was assigned if the study did not meet criteria. Quality score was calculated by summing up all the individual item scores. Studies could receive up to 9 points on the basis of criteria. The score for population and outcome assessment was 1, that for study design was 4 and that for data analysis and presentation was 3 (Table 2). Studies were classified into high, moderate or low quality on the basis of their methodological quality scores and statistical analysis methods (upper part of Table 3). None of the studies were excluded on the basis of quality assessment results.

**TABLE 2:** Criteria for the assessment of the methodological quality of observational studies.

Aspect	Criterion	Score
Population	① Sample size ≥ 50 and participation rate ≥ 80%	1
Participant selection	② For cohort/longitudinal and case–control studies, selected subjects were representative of the study population; for cross‐sectional studies, the inclusion criteria of caregivers were clearly defined, and subjects and settings were described in detail	1
Study design	③ Cohort/longitudinal design with the follow‐up duration reported	2
	④ Case–control or cross‐sectional design	1
	⑤ Withdrawals ≤ 20%	1
Measurement of outcome	⑥ A validated FCR measurement tool was used	1
Statistical analysis and presentation	⑦ Appropriate statistical analyses were used	1
	⑧ Multiple regression/multivariate analysis was performed	1
	⑨ Frequencies of caregiver FCR were reported	1

**TABLE 3:** Criteria for the assessment of the quality level of studies and best‐evidence synthesis.

Item	Level	Subject selection
Quality level of included studies	High	Multivariate analysis was performed and quality score ≥ 7
	Moderate	Multivariate analysis was performed and quality score < 7 or no multivariate analysis was performed and quality score > 5
	Low	No multivariate analysis was performed and quality score ≤ 5
Level of evidence	Strong	Minimum of three high‐quality studies with generally consistent findings
	Moderate	Minimum of two moderate‐quality studies with generally consistent findings
	Limited	Minimum of one low‐quality study with generally consistent findings
	Conflicting	Converse findings in > 25% of the studies
	None	No studies could be found

### 2.6 Data Extraction

Data extraction was performed independently by two reviewers using a predesigned standardised form in Word (Microsoft) to extract key information from relevant studies. Any discrepancies between the two reviewers were resolved by discussion with other reviewers. Additionally, the authors of the included studies were contacted to obtain any unclear or missing data. The information extracted included the characteristics of the study (i.e., authors, publication year, country, study design and sample size), profile of caregivers (i.e., age, gender and relationship to the women with breast cancer), clinical characteristics of the women with breast cancer (i.e., cancer stage) and information related to caregiver FCR (i.e., the instrument used, prevalence, mean [*M*] and standard deviation [SD] of FCR and factors identified to be associated with caregiver FCR).

### 2.7 Data Analysis

Given the heterogeneity of the included studies and independent risk factors, we were unable to perform a meta‐analysis directly. Instead, study design and sample characteristics were described. In addition to assessing the quality of the studies, we graded the body of evidence. Therefore, we summarised the results by using the best‐evidence synthesis model (lower part of Table 3) to classify potential risk factors. This approach is less common than others but is increasingly recognised as pertinent because it provides a conclusion that incorporates the quality of studies and their outcomes (Gomes and Higginson 2006; Lievense et al. 2002; Xing et al. 2013; Zhou et al. 2013). ‘Strong evidence’ means that further study is very unlikely to change our confidence in the estimate of the effect. ‘Moderate evidence’ indicates that further research is likely to have an effect on our confidence in the estimate of the effect and may change results. ‘Limited evidence’ means that further research is very likely to change results. ‘Conflicting evidence’ means that any estimate of effect is very uncertain. ‘No evidence’ means that no statistically analysed or discussed factors are presented. We also conducted subgroup analysis based on caregiver type (spouse/partner vs. other type of caregivers).

### 2.8 Meta‐Analysis

Review Manager Version 5.3 was used to perform statistical analyses. MD with 95% CIs was used to calculate generic inverse variance measured with different tools across studies. The prevalence of FCR was extracted from each study. Standard error (SE) was calculated by the formula (*P* × [1 − *P*]/*n*)^0.5^, where *n* represents the sample size in the original studies. FCR prevalence and SE were then input into Revman 5.3. Initially, a fixed‐effects model was used in the data analysis. Heterogeneity across studies was assessed by using *I* ^2^ statistics. We considered *p* < 0.10 statistically significant for the chi‐square test, indicating heterogeneity. *I* ^2^ values of 0%–40% were regarded as not important heterogeneity, those of 30%–60% as moderate heterogeneity, those of 50%–90% as substantial heterogeneity and those of 75%–100% as considerable heterogeneity (Higgins 2019). If the *I* ^2^ value exceeded 50%, the random‐effects model was chosen. Sensitivity analysis was performed by excluding one study at a time to examine whether the results could have been influenced by a single study. Publication bias was not evaluated because only five studies were included in the meta‐analysis (Dalton et al. 2016).

## 3 Results

### 3.1 Search Results

**FIGURE 1:**
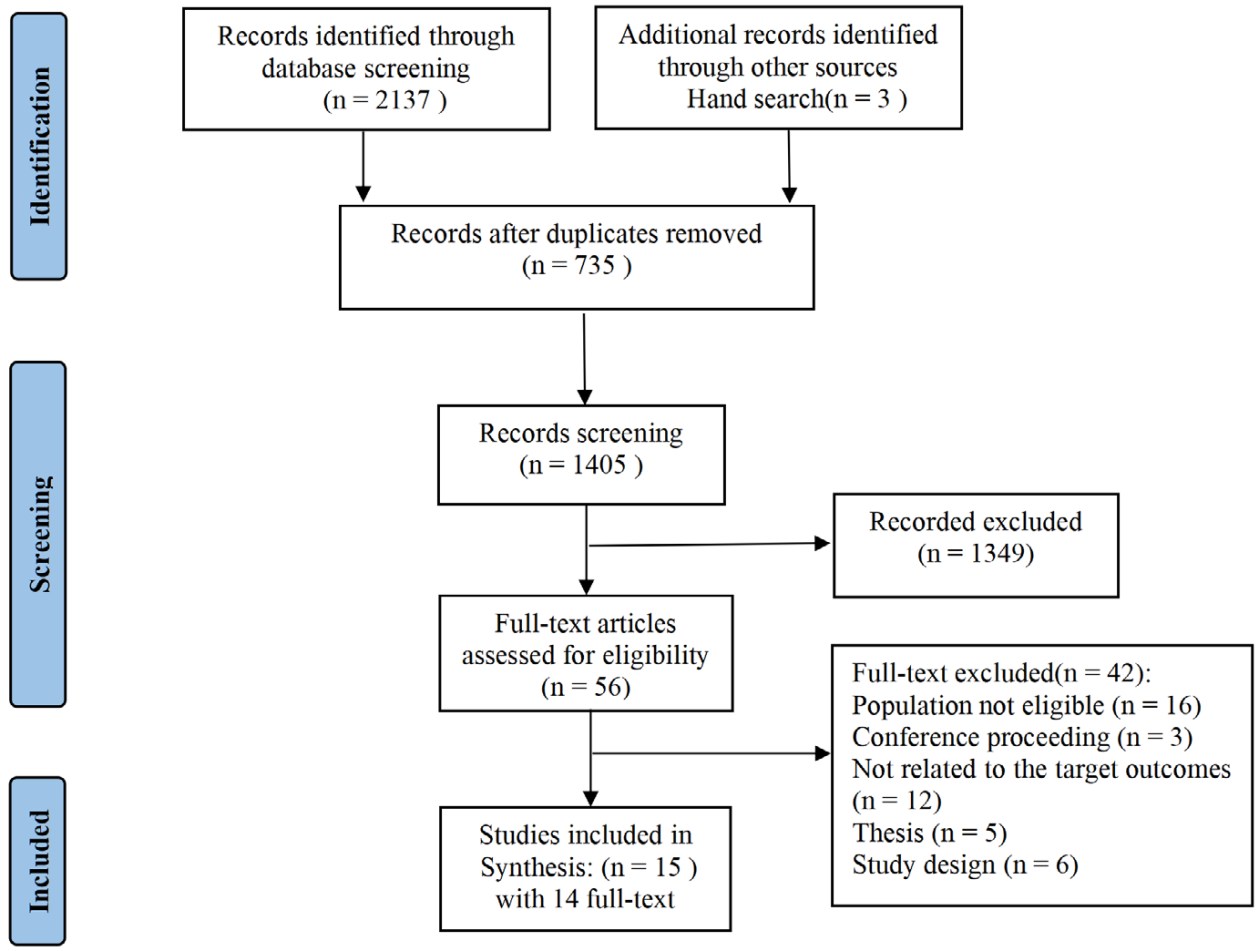
Flow diagram of the study selection procedure. [Colour figure can be viewed at wileyonlinelibrary.com]

We identified 2137 studies in the initial literature search of eight databases. In addition, we retrieved three records through manual searching. After we removed 735 duplicates, we reviewed the titles and abstracts of 1405 papers, with 56 full‐text articles reviewed for eligibility. A total of 14 papers reporting 15 studies were included in the review (Figure 1). One paper included two studies with different study designs, sample sizes and assessment tools (Soriano, Pasipanodya, et al. 2018).

### 3.2 Study Characteristics

Of the included 15 studies, five were conducted in China (Wang et al. 2021; Xia et al. 2023; Xu et al. 2019; Zhang, Hu et al. 2023; Zhang, Yu et al. 2023), nine in the United States (Boehmer et al. 2016; Cohee et al. 2017; Janz et al. 2016; Perndorfer et al. 2019; Soriano et al. 2021; Soriano, Perndorf et al. 2018; Soriano, Pasipanodya et al. 2018; Soriano et al. 2019) and one in Germany (Muldbücker et al. 2021). All the reviewed studies were published between 2016 and 2023 (Table 4). These 15 studies involved 2461 eligible caregivers with sample sizes ranging from 46 to 510. The participation rate of the studies ranged from 0.12 to 0.92. Almost all the studies included spouses or partners as caregivers. However, one study included other types of caregivers, such as other family members and friends (Boehmer et al. 2016). Five studies (Boehmer et al. 2016; Soriano et al. 2021; Soriano, Pasipanodya et al. 2018; Soriano et al. 2019; Janz et al. 2016) did not only include male partners but also female partners due to sex orientation. Three studies (Cohee et al. 2017; Perndorfer et al. 2019; Soriano, Perndorfer et al. 2018) did not mention the genders of the caregivers. One study included a mixed cancer population but performed a subgroup analysis for breast cancer (Muldbücker et al. 2021). All women with breast cancer were at cancer stages 0–IV (Table 4).

**TABLE 4:** Characteristics of the included studies.

Author (year)	Country	Study design	Sample size	Final sample size	Participation rate	Age (*M* ± SD)	Gender	Relationship to women with breast cancer	Cancer stage
Xu et al. (2019)	China	Longitudinal	217	54	0.25	22–65	Male	Spouses	I–III
Wang et al. (2021)	China	Cross‐sectional	237	214	0.90	≥ 25	Male	Spouse	/
Xia et al. (2023)	China	Cross‐sectional	274	253	0.92	/	Male	Spouse	I–IV
Zhang, Hu et al. (2023)	China	Cross‐sectional	350	332	0.95	25–74 (51.45 ± 8.76)	Male	Spouse	I–IV
Zhang, Yu et al. (2023)	China	Longitudinal	224	192	0.86	18–75	Male	Spouse	I–IV
Boehmer et al. (2016)	The United States	Cross sectional	297	167 (male = 43, female = 124)	0.56	Caregiver of heterosexual survivor: 62.4 ± 8, caregiver of sexual minority survivor: 55.8 ± 9.3	Male and female	Spouse, partners, other family members and friends	In situ (ductal carcinoma) + I–III + missing 1
Janz et al. (2016)	The United States	Cross sectional	774	510	0.66	/	Inclusion criteria not limited by partner's gender	Partners	0–III
Cohee et al. (2017)	The United States	Cross sectional	397	222	0.37	30–75	/	Partners	I–IIIa
Soriano, Perndorfer et al. (2018)	The United States	Longitudinal	110	79	0.72	/	/	Spouses	0–III
Soriano, Pasipanodya et al. (2018) (1)	The United States	Cross sectional (Study 1)	122	46	0.38	54.57 ± 13.31	Male	Spouses	0–IIIa
Soriano, Pasipanodya et al. (2018) (2)	The United States	Longitudinal study (Study 2)	463	72 (male = 70, female = 2)	0.16	59.49 ± 10.34	Male and female	Partners	0–IIIa
Perndorfer et al. (2019)	The United States	Longitudinal	271	69	0.25	58 ± 10	/	Partners	0–IIIa
Soriano et al. (2019)	The United States	Longitudinal	353	57	0.12	60 ± 10	Male and female	Partners	0–IIIa
Soriano et al. (2021)	The United States	Longitudinal	270	79 (male = 77, female = 2)	0.29	59.38 ± 10.39	Male and female	Partners	0–IIIa
Muldbücker et al. (2021)	Germany	Cross sectional	/	115	/	52.9 ± 11.0	Male	Partners	/

### 3.3 Quality of Studies

Of the 15 included studies, three (one had a quality score of 8 and two had a quality score of 7) were assessed to be of high quality. Four studies with a quality score of 6, seven studies with a quality score of 5 and one study with a quality of 4 were rated as of moderate quality. Therefore, three studies had high quality and 12 studies had moderate quality (Table 5).

**TABLE 5:** Quality assessment results of the 16 reviewed studies in 15 published articles.

Author (year)	①	②	③	④	⑤	⑥	⑦	⑧	⑨	Total score	Level
Xu et al. (2019)	0	1	2	0	0	0	0	1	0	4	Moderate
Wang et al. (2021)	1	1	0	1	0	1	1	1	1	7	High
Xia et al. (2023)	1	1	0	1	0	1	0	1	1	6	Moderate
Zhang, Hu et al. (2023)	1	1	0	1	0	1	0	1	0	5	Moderate
Zhang, Yu et al. (2023)	1	1	2	0	1	1	1	1	0	8	High
Boehmer et al. (2016)	0	1	0	1	0	1	1	1	0	5	Moderate
Janz et al. (2016)	0	1	0	1	0	0	1	1	1	5	Moderate
Cohee et al. (2017)	0	1	0	1	0	1	1	1	0	5	Moderate
Soriano, Perndorfer et al. (2018)	0	1	2	0	1	1	0	1	0	6	Moderate
Soriano, Pasipanodya et al. (2018) (①)	0	1	0	1	0	1	1	1	0	5	Moderate
Soriano, Pasipanodya et al. (2018) (②)	0	1	2	0	0	1	1	1	0	6	Moderate
Perndorfer et al. (2019)	0	1	2	0	1	1	0	1	1	7	High
Soriano et al. (2019)	0	1	2	0	0	1	0	1	0	5	Moderate
Soriano et al. (2021)	0	1	2	0	0	1	0	1	1	6	Moderate
Muldbücker et al. (2021)	0	1	0	1	0	1	1	1	0	5	Moderate

*Note:* ① Sample size ≥ 50 and participation rate ≥ 80%. ② For cohort and case–control studies: selected subjects were representative of the study population. For cross‐sectional studies: inclusion criteria for caregivers were clearly defined, and subjects and settings were described in detail. ③ Cohort/longitudinal design with the duration of follow‐up reported. ④ Case–control or cross‐sectional design. ⑤ Withdrawals ≤ 20%. ⑥ A validated FCR assessment instrument was used. ⑦ Appropriate statistical analyses were used. ⑧ Multiple regression/multivariate analysis was performed. ⑨ Frequencies of caregiver FCR were reported.

### 3.4 Prevalence and Severity of Caregiver FCR

On the basis of five studies that had reported prevalence data, the proportion of caregivers with a significant level of FCR varied from 19% to 64.95% (Janz et al. 2016; Perndorfer et al. 2019; Soriano et al. 2021; Wang et al. 2021; Xia et al. 2023). One cross‐sectional study and two longitudinal studies conducted in the United States reported that between 19% and 42.3% of partners of women with breast cancer had clinical FCR (Janz et al. 2016; Perndorfer et al. 2019; Soriano et al. 2021). Two cross‐sectional studies conducted in China also reported that between 51.40% and 64.95% of partners of women with breast cancer had clinical FCR (Wang et al. 2021; Xia et al. 2023). The mean and/or SD of FCR scores were reported in the majority of the studies (Boehmer et al. 2016; Cohee et al. 2017; Muldbücker et al. 2021; Perndorfer et al. 2019; Soriano et al. 2021; Soriano, Pasipanodya, et al. 2018; Soriano et al. 2019; Xia et al. 2023; Zhang, Hu et al. 2023). One study identified and classified the trajectory of FCR into three categories, namely, late remission (32.8%), slow decline (50.5%) and persistent fear (16.7%) (Zhang, Yu et al. 2023). One study (Soriano et al. 2021) used two measurement tools, whereas the other 14 studies used one tool to measure FCR in caregivers. Across the studies, FCR was measured by using four different validated measurement tools, including Fear of Progression Questionnaire—Short Form for Partner (Muldbücker et al. 2021; Wang et al. 2021; Xia et al. 2023; Zhang, Hu et al. 2023; Zhang, Yu et al. 2023), 22‐item Fear of Cancer Recurrence Questionnaire (Boehmer et al. 2016), Concerns About Recurrence Scale (Cohee et al. 2017; Soriano et al. 2021) and Fear of Cancer Recurrence Inventory (Perndorfer et al. 2019; Soriano, Pasipanodya et al. 2018; Soriano, Perndorfer, et al. 2018; Soriano et al. 2019). In two studies, the FCR assessment tool was used without validation, with one using five items covering worries about the future, death, recurrence of cancer, diffusion of disease and treatment of cancer (Xu et al. 2019). The other study measured worry by one item ‘how often they worry about the possibility that their spouse or partner's breast cancer might recur’ on a 5‐point Likert scale (‘not at all’ to ‘a lot’) (Janz et al. 2016) (Table 6).

**TABLE 6:** Outcome assessment tool and results.

Author (year)	Assessment tool	Validated	Mean ± SD of FCR	Prevalence of FCR
Xu et al. (2019)	Adapted five items widely used in prior research: worries about the future, death, recurrence of cancer, diffusion of disease and treatment of cancer	×	19.82 ± 17.77	/
Xia et al. (2023)	FoP‐Q‐SF/P	√	34.3 ± 7.1	51.40%
Wang et al. (2021)	FoP‐Q‐SF/P	√	36.68 ± 10.21	64.95% (*n* = 139) had clinical FCR
Zhang, Hu et al. (2023)	FoP‐Q‐SF/P	√	18.48 ± 5.23	/
Zhang, Yu et al. (2023)	FoP‐Q‐SF/P	√	/	Late remission group (*n* = 63), slow descent group (*n* = 97), stable fear group (*n* = 32)
Boehmer et al. (2016)	22‐item Fear of Cancer Recurrence Questionnaire	√	Caregiver of heterosexual survivor: 75.2 ± 13.6, caregiver of sexual minority survivor: 71.8 ± 16.5	/
Janz et al. (2016)	Not validated; ‘how often they worry about the possibility that their spouse or partner's breast cancer might recur’ on a 5‐point Likert scale	×	/	47.1% (*n* = 212) reported worry about recurrence
Cohee et al. (2017)	CARS	√	11.79 ± 5.02	/
Soriano, Perndorfer et al. (2018)	Six items from the Distress, Insight and Severity subscales of the FCRI	√	/	/
Soriano, Pasipanodya et al. (2018) (①)	Four‐item Overall Fear subscale of CARS	√	*Mean*: 3.18 Between‐person SD: 0.17	/
Soriano, Pasipanodya et al. (2018) (②)	Used six items culled from the severity, distress and insight subscales of FCRI	√	*Mean*: 1.51 Between‐person SD: 2.43 Within‐person SD: 2.07	/
Perndorfer et al. (2019)	Six items from the distress, insight and severity subscales of FCRI	√	Mean: 1.43	39% (*n* = 27) reported clinical levels on the global FCRI‐severity subscale
Soriano et al. (2019)	Six of the highest‐loading items from FCRI	√	0.96 ± 1.78	/
Soriano et al. (2021)	Severity and distress subscales of FCRI; the overall fear subscale of the CARS	√	/	19% reported clinical levels of FCR
Muldbücker et al. (2021)	FoP‐Q‐SF/P	√	30.07 ± 8.2	/

Abbreviations: FoP‐Q‐SF/P: Fear of Progression Questionnaire—Short Form/Partner; CARS: Concerns about Recurrence Scale; FCRI: Fear of Cancer Recurrence Inventory.

### 3.5 Associated Factors of FCR

Twenty‐nine factors, amongst which 10 were patient related and 19 were caregiver related, were identified to be associated with high FCR in caregivers of women with breast cancer. We further classified the 10 patient‐related associated factors into five groups, namely, medical information (i.e., time since diagnosis and treatment), demographic information (i.e., comorbidities and age), psychological factors (i.e., patient's FCR), psychosocial factors (i.e., responsiveness, social constraints and protective buffering) and relationship (i.e., couple communication and marital commitment) and the 19 caregiver‐related associated factors into four groups, namely, demographic information (i.e., age, caregiving time, residence, comorbidities, race and as primary caregiver), psychological factors (i.e., resilience, cognitive processing, perception of positive information, depression, threat sensitivity and momentary negative affect), psychosocial factors (i.e., social support, social constraints and protective buffering) and relationship (i.e., relationship quality, responsiveness, communication and marital commitment).

On the basis of the synthesis, five moderate level factors were identified, with one patient‐related factor (i.e., communication) and four caregiver‐related factors (i.e., resilience, social constraints, protective buffering and communication). Fifteen factors, with four patient‐related and 11 caregiver‐related factors, were graded at a limited level. Nine factors, with five patient‐related and four caregiver‐related factors, were graded at a conflicting level (Table 7).

**TABLE 7:** Summary of factors associated with high caregiver FCR.

Population	Group	Factors	Level of evidence	Modifiable	No. of caregivers in high‐quality studies	No. of caregivers in moderate‐quality studies
Patients	Medical information	Time since diagnosis	Conflicting	×	/	282 (Boehmer et al. 2016; Muldbücker et al. 2021)
		Treatment	Conflicting	×	/	677 (Boehmer et al. 2016; Janz et al. 2016)
	Demographic information	Comorbidities	Limited	×	/	167 (Boehmer et al. 2016)
		Age	Conflicting	×	/	118 (Soriano, Pasipanodya et al. 2018)
	Psychological factors	FCR	Conflicting	√		899 (Boehmer et al. 2016; Janz et al. 2016; Cohee et al. 2017)
	Psychosocial factors	Responsiveness	Limited	√		79 (Soriano, Perndorfer et al. 2018)
		Social constraints	Conflicting	√	/	118 (Soriano, Pasipanodya et al. 2018)
		Protective buffering	Limited	√	69 (Perndorfer et al. 2019)	/
	Relationship	Communication	Moderate	√		465 (Soriano, Perndorfer et al. 2018; Xu et al. 2019; Zhang, Hu et al. 2023)
		Marital commitment	Limited	√		253 (Xia et al. 2023)
Caregivers	Demographic information	Age	Conflicting	×	214 (Wang et al. 2021)	625 (Janz et al. 2016; Muldbücker et al. 2021)
		Caregiving time	Limited	×	214 (Wang et al. 2021)	/
		Residence	Limited	×	192 (Zhang, Yu et al. 2023)	/
		Comorbidities	Conflicting	×	192 (Zhang, Yu et al. 2023)	510 (Janz et al. 2016)
		Race	Limited	×	/	510 (Janz et al. 2016)
		As primary caregiver	Limited	×	192 (Zhang, Yu et al. 2023)	/

	Psychological factors	Resilience	Moderate	√	406 (Wang et al. 2021; Zhang, Yu et al. 2023)	/
		Cognitive Processing	Limited	√	/	222 (Cohee et al. 2017)
		Perceptions of positive information	Limited	×	/	54 (Xu et al. 2019)
		Depression	Limited	√	/	115 (Muldbücker et al. 2021)
		Threat sensitivity	Limited	√	/	57 (Soriano et al. 2019)
		Momentary negative affect	Limited	√	/	72 (Soriano, Pasipanodya et al. 2018)
	Psychosocial factors	Social support	Conflicting	√	192 (Zhang, Yu et al. 2023)	677 (Boehmer et al. 2016; Janz et al. 2016)
		Social constraints	Moderate	√	/	419 (Cohee et al. 2017; Soriano et al. 2021; Soriano, Pasipanodya et al. 2018)
		Protective buffering	Moderate	√	69 (Perndorfer et al. 2019)	79 (Soriano et al. 2021)
	Relationship	Relationship quality	Conflicting	√	/	312 (Soriano, Pasipanodya et al. 2018; Soriano et al. 2021; Muldbücker et al. 2021)
		Responsiveness	Limited	√	/	79 (Soriano, Perndorfer et al. 2018)
		Communication	Moderate	√	/	465 (Soriano, Perndorfer et al. 2018, Xu et al. 2019; Zhang, Hu et al. 2023)
Marital commitment		Limited	√	/	253 (Xia et al. 2023)

#### 3.5.1 Patient‐Related Associated Factors

One patient‐related factor, insufficient couple communication (Soriano, Perndorfer et al. 2018; Xu et al. 2019; Zhang, Hu et al. 2023), was a significant factor associated with high caregiver FCR with moderate evidence. Four patient‐related associated factors (i.e., comorbidities, responsiveness and protective buffering) were significant factors associated with high caregiver FCR with limited‐level evidence. The evidence of the associations of the other five patient‐related associated factors with high FCR was conflicting because it was supported by two or three studies (Table 7).

#### 3.5.2 Caregiver‐Related Associated Factors

Four caregiver‐related associated factors had moderate evidence: one psychological factor (i.e., resilience), two psychosocial factors (social constraints and protective buffering) and one relationship factor (i.e., communication). The results showed that a low level of resilience (Wang et al. 2021; Zhang, Yu et al. 2023), high social constraints (Cohee et al. 2017; Soriano et al. 2021; Soriano, Pasipanodya et al. 2018), high protective buffering (Perndorfer et al. 2019; Soriano et al. 2021) and insufficient couple communication (Soriano, Perndorfer et al. 2018; Xu et al. 2019; Zhang, Hu et al. 2023) were significantly associated with high FCR in caregivers. Eleven caregiver‐related associated factors had limited evidence because they were supported by only one high‐ or moderate‐quality study. The evidence for the remaining four factors (i.e., age, comorbidities, social support and relationship quality) with FCR in caregivers was graded as conflicting because they were supported by two to four studies with significant and insignificant *p* values (*p* > 0.05) (Table 7).

#### 3.5.3 Subgroup Analysis by Caregiver Type

Given that all included works, except the study by Boehmer et al. (2016), included spouse/partners as the target population, we conducted a subgroup analysis by excluding this study. The results on the number and level of evidence for 19 caregiver‐related factors remained the same in the subgroup analysis. However, the evidence for three of the 10 patient‐related associated factors was changed, with time since diagnosis and comorbidity factors changing from limited to no evidence and treatment changing from conflicting to limited. Therefore, the number of patient‐related associated factors changed from 10 to eight: one moderate (communication), four limited (treatment, protective buffering, responsiveness and marital commitment) and three conflicting (age, social constraints and FCR) in the subgroup analysis (Table 8).

**TABLE 8:** Patient‐related factors in subgroup analysis by type of caregiver.

Population	Group	Factors	Level of evidence	Modifiable	No. of caregivers in high‐quality studies	No. of caregivers in moderate‐quality studies
Patients	Medical information	Time since diagnosis	None	×	/	
		Treatment	Limited	×	/	510 (Janz et al. 2016)
	Demographic information	Comorbidities	None	×	/	
		Age	Conflicting	×	/	118 (Soriano, Pasipanodya et al. 2018)
	Psychological factors	FCR	Conflicting	√		732 (Janz et al. 2016; Cohee et al. 2017)
	Psychosocial factors	Responsiveness	Limited	√		79 (Soriano, Perndorfer et al. 2018)
		Social constraints	Conflicting	√	/	118 (Soriano, Pasipanodya et al. 2018)
		Protective buffering	Limited	√	69 (Perndorfer et al. 2019)	/
	Relationship	Communication	Moderate	√		465 (Soriano, Perndorfer et al. 2018; Xu et al. 2019; Zhang, Hu et al. 2023)
		Marital commitment	Limited	√		253 (Xia et al. 2023)

### 3.6 Meta‐Analysis on the Prevalence of Caregiver FCR

**FIGURE 2:**
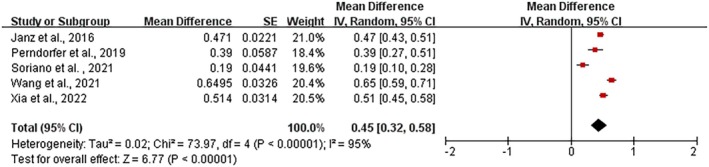
Forest plot of prevalence for caregiver FCR. [Colour figure can be viewed at wileyonlinelibrary.com]

Five studies were included to determine FCR prevalence. The combined FCR prevalence of five studies was 45% (95% CI: 0.43–0.51) and showed substantial heterogeneity (*I* ^2^ = 93%, *p* < 0.00001) (Figure 2).

### 3.7 Sensitivity Analysis

In sensitivity analysis, pooled prevalence fluctuated between 39% (95% CI: [0.27, 0.52]) and 51% (95% CI: [0.42, 0.61]) when one study was excluded at a time for comparison. The pooled effect sizes remained significant when a study was excluded, indicating that the overall significant result was robust and not influenced by any study.

## 4 Discussion

Caregivers live with a high degree of uncertainty and reported FCR levels at least as high as those reported by patients with cancer, with some caregivers reporting FCR levels even higher than patients (Webb et al. 2023). The prevalence of FCR in our systematic review varied from 19% to 64.95% (Janz et al. 2016; Perndorfer et al. 2019; Soriano et al. 2021; Wang et al. 2021; Xia et al. 2023). Our meta‐analysis showed that the prevalence of caregiver FCR is 45%. Our systematic review summarised the factors associated with high FCR levels in caregivers of women with breast cancer. Given the paucity of primary studies on FCR levels in caregivers of women with breast cancer and the high degree of variability in terms of investigated factors, many of the factors associated with high FCR were of limited evidence. The evaluation of the current evidence identified five moderate‐level factors, 15 limited‐level factors and nine conflicting‐level factors associated with high FCR levels in caregivers. Of the five moderate‐level evidence factors, one was a patient‐related factor (i.e., communication) and four were caregiver‐related factors (resilience, social constraints, protective buffering and communication).

### 4.1 Patient‐Related Factors

One moderate‐level factor, poor couple communication, was a significant factor associated with high caregiver FCR. Women's expressions in response to negative information are readily perceived by their caregivers, thus leading to an increase in their partners' FCR (Xu et al. 2019). Obstacles may also exist between women with breast cancer and their caregivers; for example, patients may hide their fragile and sensitive emotions from their families or avoid taking the initiative to talk to others about their negative emotions, resulting in insufficient communication. Consequently, the pressure on caregivers cannot be properly relieved, and their level of concern about disease progression and prognosis gradually increases (Zhang, Hu et al. 2023). This situation may finally lead to high FCR level in caregivers (Zhang, Hu et al. 2023).

Other four patient‐related associated factors, that is, comorbidities, responsiveness, protective buffering and marital commitment, were significant factors associated with high caregiver FCR with limited evidence. Patients with numerous comorbidities are likely too weak to fight cancer, contributing to high FCR in caregivers. The responsiveness of the patients affected caregiver FCR as well. Caregivers disclose positive events to patients, and whether patients respond with interest or enthusiasm can decrease their FCR levels. In caregivers, FCR was buffered when patients were perceived as responsive and interested. Protective buffering is defined as efforts to protect one's partner from distress and burden by hiding or denying cancer‐related concerns and yielding to the partner to avoid disagreements (Coyne and Smith 1991). Patients and their caregivers perform protective buffering. They avoid discussing cancer or their worries to protect and reduce each other's distress and burden. They find sharing cancer‐related concerns with friends and family stressful because they do not want to upset them (Lai et al. 2019; Şengün İnan and Üstün 2018). Women with breast cancer try to protect their family members by not talking about their concerns and carry their burden alone. Caregivers experienced high FCR when patients had good marital commitment. This result may be, at least partly, obtained because couples with better relationships care more about the effect of breast cancer on women's health than those with poorer relationships, resulting in high caregiver FCR.

### 4.2 Caregiver‐Related Factors

Four moderate‐level factors were related to caregivers. One moderate‐level psychological factor, namely, low level of resilience in caregivers, was correlated with high caregiver FCR. Resilience included psychological and family resilience. The level of FCR in partners with high psychological resilience is likely to decrease. Studies have shown that psychological resilience could buffer the adverse effects caused by negative life events and is conducive to accepting the fact of illness, facing disease with a peaceful mind and improving mental health (Zhang, Yu et al. 2023). Family resilience was negatively correlated with FCR in partners. Families with high levels of family resilience were likely to respond positively to cancer events (Wang et al. 2021).

Two caregiver‐related psychosocial factors with a moderate level of evidence included social constraints and protective buffering and were correlated with caregiver FCR. Social constraint was a statistically significant predictor of high FCR in partners (Soriano et al. 2021; Soriano, Pasipanodya, et al. 2018). Partners may have social constraints on disclosure due to numerous reasons. First, some partners did not talk about their experience because they thought it was not part of their gender role (Hilton et al. 2000; Zahlis and Lewis 2010). Furthermore, similar to protective buffering in women, partners may want to prevent family members from worry, avoid conflict or protect women's feelings (Zahlis and Lewis 2010). Moreover, an unsupportive social environment, such as myths, misconceptions and beliefs about breast cancer, invariably engenders gossip and social stigmatisation (Boamah Mensah et al. 2021; e Silva et al. 2010). All these reasons may cause partners to feel constrained to express their thoughts, feelings or concerns towards their loved ones living with breast cancer. In addition, cognitive processing mediated the relationship between social constraints and FCR in partners. These findings suggest that when caregivers feel constrained to talk about breast cancer, they are unable to process the trauma caused by the disease (Cohee et al. 2017).

One relationship‐related factor, insufficient communication, was identified as a moderate‐level factor for caregiver FCR. Low likelihood to communicate entails high FCR in caregivers likely because the relationship and intimacy between couples may be enhanced by communicating and disclosing each other's feeling and thoughts, which in turn enable couples to adapt to cancer and hence reduce FCR (Manne and Badr 2008). Caregiver FCR may affect couples' decision‐making regarding fertility preservation. Survival is likely the priority of most women with breast cancer and their caregivers, and ovarian stimulation carries a high risk and could affect recurrence rates (Lee et al. 2011). Breast cancer treatment may affect fertility. Families without children aspire to have a complete family and believe that children are an important hub for maintaining the family. Therefore, providing fertility preservation and reproductive counselling is important, and the provision of fertility psychoeducation and psychosocial counselling for emotional support can assist with fertility treatment decision‐making (Logan and Anazodo 2019).

Nine conflicting factors, amongst which five were patient‐related factors and four were caregiver‐related, and 15 factors had limited evidence. Additional studies are needed to examine the relationship between those factors with caregiver FCR level.

## 5 Strengths and Limitations

We conducted a comprehensive search, and the included studies were all of high or moderate quality with cross‐sectional and longitudinal designs. Best‐evidence synthesis is useful for making clinical recommendations. Therefore, the results of this systematic review should be considered valid. Furthermore, we identified studies from western and Asia countries and included caregivers of heterosexual and sexual minority patients. This approach greatly enhanced the generalizability of the findings of our review. However, our study is not without limitations. The use of different measurement tools of caregiver FCR across the included studies precludes the comparison of caregiver FCR amongst studies. Moreover, nearly all the caregivers were partners or spouses of women with breast cancer. Only one work (Boehmer et al. 2016) included other types of caregivers. Therefore, the evidence generated from this systematic review is limited to partners and cannot be extended other types of caregivers.

## 6 Conclusions

The prevalence of FCR was 45%. Our systematic review provided a summary of factors associated with FCR in caregivers of women with breast cancer. A total of 29 factors were identified to be associated with high FCR in caregivers. Of these factors, five were supported by moderate evidence, 1 were supported by limited evidence and nine were supported by conflicting evidence. In general, caregivers with a lower level of resilience, more social constraints, more protective buffering and insufficient communication tended to experience higher FCR levels than those without. Furthermore, caregivers tended to experience high FCR if women with breast cancer had low communication.

### 6.1 Relevance to Clinical Practice

Identifying factors contributing to FCR in caregivers of women with breast cancer is important to develop interventions for caregivers most in need and reduce adverse health outcomes related to caregiver FCR. Future research may start from modifiable factors for intervention development. For example, communication, social constraints and protective buffering were common modifiable associated factors of FCR in women with breast cancer and their caregivers. When people have social constraints on the disclosure of disease‐related concerns or issues, they encounter barriers in expressing their emotions and thoughts. Such a situation may inhibit cognitive processing and exacerbate emotional distress. Protective buffering occurs when individuals avoid discussing their cancer experience to protect and reduce one's distress and burden. Intervention focusing on disclosure or communication between women with breast cancer and their caregivers is promising for reducing social constraints and promoting protective buffering, which can in turn lead to a reduction in FCR in both parties.

The associated factors examined by our systematic review provides some evidence for identifying caregivers who are at high risk of high FCR. However, additional high‐quality studies with wide coverages of the studied factors and appropriate statistical analysis strategies are needed to clarify and determine the estimates of the effects of individual factors. Furthermore, given the differences in social patterns, culture, value and family structure across different countries, we encourage other researchers in other countries to conduct this kind of study because we included studies only from China, the United States and Germany.

## Conflicts of Interest

The authors declare no conflicts of interest.

## Supporting information


**Data S1:** Supporting Information (PRISMA 2020 checklist).

## Data Availability

The data that support the findings of this study are available on request from the corresponding author. The data are not publicly available due to privacy or ethical restrictions.
